# Carcinogenesis and Metastasis in Liver: Cell Physiological Basis

**DOI:** 10.3390/cancers11111731

**Published:** 2019-11-05

**Authors:** Anna Rossetto, Valli De Re, Agostino Steffan, Matteo Ravaioli, Gianmaria Miolo, Patrizia Leone, Vito Racanelli, Alessandro Uzzau, Umberto Baccarani, Matteo Cescon

**Affiliations:** 1 Department of Organ Insufficiency and Transplantation, General Surgery and Transplantation, University Hospital of Bologna, Policlinico S. Orsola-Malpighi, 40138 Bologna, Italy; matteo.ravaioli@aosp.bo.it (M.R.); matteo.cescon@aosp.bo.it (M.C.); 2 Immunopatologia e Biomarcatori Oncologici/Bio-proteomics Facility, Centro di Riferimento Oncologico di Aviano (CRO), IRCCS, 33081 Aviano, Italy; asteffan@cro.it; 3 Department of Medical Oncology, Centro di Riferimento Oncologico di Aviano (CRO), IRCCS, 33081 Aviano, Italy; gmiolo@cro.it; 4 Department of Biomedical Sciences and Human Oncology, G. Baccelli Section of Internal Medicine, University of Bari Medical School, 70124 Bari, Italy; patrizia.leone@uniba.it (P.L.); vito.racanelli1@uniba.it (V.R.); 5 Program of Oncology Surgery, Dipartimento di Area Medica, University of Udine, 33100 Udine, Italy; alessandro.uzzau@uniud.it; 6 Surgery and Transplantation, Dipartimento di Area Medica, University of Udine, 33100 Udine, Italy; umberto.baccarani@uniud.it

**Keywords:** hepatocellular carcinoma, nonalcoholic fatty liver disease, nonalcoholic steatohepatitis, hepatic stellate cells, myofibroblasts, VEGF, immunity, circadian homeostasis, cortisol

## Abstract

Hepatocellular carcinoma (HCC) incidence is rising. This paper summarises the current state of knowledge and recent discoveries in the cellular and physiological mechanisms leading to the development of liver cancer, especially HCC, and liver metastases. After reviewing normal hepatic cytoarchitecture and immunological characteristics, the paper addresses the pathophysiological factors that cause liver damage and predispose to neoplasia. Particular attention is given to chronic liver diseases, metabolic syndrome and the impact of altered gut microbiota, disrupted circadian rhythm and psychological stress. Improved knowledge of the multifactorial aetiology of HCC has important implications for the prevention and treatment of this cancer and of liver metastases in general.

## 1. Introduction

Hepatocellular carcinoma (HCC) is an aggressive tumour of the liver. Among all cancers, HCC is one of the fastest growing causes of death globally [1]. HCC is often diagnosed when it has reached an advanced stage, so it is often impossible to offer treatment with curative intent. Generally, HCC arises in a liver that is compromised due to chronic injury or inflammation subsequent to chronic infection with hepatitis B virus or hepatitis C virus (HCV). Vaccination against hepatitis B virus has been shown to reduce HCC incidence rates in children [2]. Eradication of HCV using new direct-acting antiviral drugs reduces the risk of HCC in patients with chronic HCV infection [3]. However, it was shown that, although after hepatitis B virus or HCV eradication the regression of fibrosis or even reversal of cirrhosis may occur, these patients might have a greater HCC risk compared to pre-cirrhotic patients, and moreover, the coexistence of a metabolic syndrome (hepatic manifestation related to hypertension, diabetes mellitus, obesity and dyslipidemia) further increases the risk for HCC development [4]. However, even after virus eradication, a large percentage of patients remain at risk of HCC, and consequently, also in the presence of a sustained virologic response, the screening programs for liver diseases are required to take this possible event into account. HCC can also develop in the context of non-infectious chronic hepatopathies such as alcoholic liver disease, non-alcoholic steatohepatitis and metabolic syndrome. The last subgroup of HCC, in particular, presents at an advanced stage, with about 25% presenting with extra-hepatic metastasis [5], as surveillance is not performed in a non-alcohol/cirrhotic liver. Thus, currently liver damage leading to F3 fibrosis, which is related to the tumour size, is now recognised as the main condition that confers a HCC risk and worsens prognosis irrespective of the aetiology [6].

When HCC develops in liver parenchyma, it is encircled by extracellular matrix, stromal cells, and secreted proteins within an immunosuppressive tumour microenvironment (TME). The TME is populated by immune cells that interact among themselves and with the tumour. The makeup of the TME depends on the biological characteristics of the neoplastic cells and on the patient’s pathophysiological conditions. The immune system’s role in promoting HCC development has long been a focus of study [7,8,9]. Particular interest has been addressed to the dual role of transforming growth factor beta (TGF-β) in regulating haematopoietic cells and promoting liver fibrosis [10]. Additionally, of interest is the role of cancer-associated fibroblasts (CAFs) in reducing the number and functional differentiation of tumour-infiltrating T cells [11]. Besides reducing the immune response, TGF-β and CAFs contribute to many neoplastic processes, including angiogenesis and tumour cell proliferation, energy metabolism reprogramming and resistance to killing.

HCC recurs after primary liver resection with curative intent in about 70% of patients [12]. The risk of recurrence in HCC is high because of the biological and morphological nature of the liver. Recurrent disease includes both intrahepatic metastasis, which usually form within the first two years of diagnosis, and de novo cancer that generally occurs later. The hepatic vascular anatomy and immunological characteristics create a pro-neoplastic niche for metastasis, while the continuing damage to the liver creates pro-neoplastic sites susceptible to secondary tumours. 

This paper reviews the normal hepatic cytoarchitecture and immunological features, and then discusses factors that, by causing liver damage, predispose to the development of HCC and liver metastases. These factors include chronic liver disease, an altered gut microbiota composition, and changes in the circadian rhythm. 

## 2. Hepatic Cytoarchitecture and Main Cell Types

**Figure 1:**
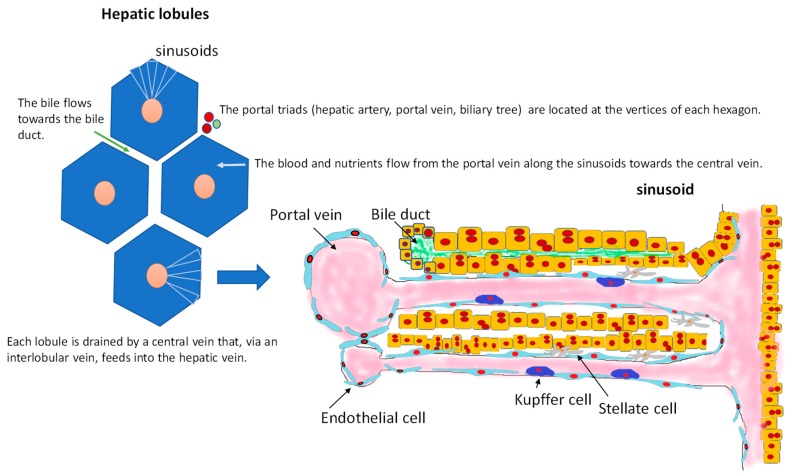
Structure of hepatic lobules. Oxygenated blood enters the lobules via a hepatic arteriole, and then flows through the sinusoids and drains into a central vein in the centrilobular region. Blood enriched in nutrients and antigens from the gastrointestinal system enters the lobules via the portal vein. This blood is filtered by Kupffer cells, which are phagocytic cells found within and below the fenestrated sinusoidal endothelium. Hepatic stellate cells (fat-storing cells that secrete matrix proteins) and antigen-presenting dendritic cells are present in the space of Disse, which separates hepatocytes (epithelial liver cells) from endothelial cells. Biliary canaliculi are located between the two cords of hepatocytes.

The main physiological functions of the liver are metabolism of nutrients absorbed by the gastrointestinal tract and detoxification of dangerous molecules present in the circulation. These functions are responsible for a delicate balance between immune tolerance and immune response, and their correct functioning requires a normal cytoarchitecture of hepatic lobules (Figure 1). This cytoarchitecture includes polarized hepatocyte and an intricate network of discontinuous capillaries (sinusoids) and bile ducts. Important cell types in the lobule are Kupffer cells, which are specialised macrophages that take up and destroy foreign material [13], and hepatic stellate cells (HSCs), which are involved in the response to liver damage [14]. HSCs are found in the space of Disse, which separates hepatocytes from sinusoids. 

The vascular endothelial growth factor (VEGF) signalling pathway regulates vascular morphogenesis, which is important for both hepatic cytoarchitecture and cancer growth. The concentration of VEGF determines the directionality of the angiogenic sprouting, and vessel extension and growth (reviewed in [15]). VEGF expression is induced by hypoxia, a condition frequently found in large tumours and associated with tumour growth. Upregulation of VEGF increases vasodilatation and blood flow and hence oxygen availability within a tumour [16]. Because VEGF is the major cytokine produced by activated HSCs, these cells are involved in maintenance of the sinusoidal endothelial cell network. HSCs control endothelial characteristics such as fenestration and permeability and regulate platelet–endothelial cell adhesion. These functions maintain the integrity of the endothelium and speed the restoration of vascular permeability after inflammatory damage [17].

HSCs are also the main cell type involved in liver fibrosis, the formation of thickened, scarred connective tissue subsequent to liver damage. Activated HSCs sustained by a profibrogenic environment differentiate into collagen-producing myofibroblasts. These cells become detectable in the liver during liver injury. They produce an abundant extracellular matrix that initially accumulates in the space of Disse [18].

Another important cell is the Kupffer cell, a self-renewing macrophage that, like the myofibroblast, accumulates in injured liver tissue. Kupffer cells recognise and clear pathogenic microbes and dying or dead host cells that arrive via the portal circulation. Depending on the local tissue environment, they adapt an M1 (pro-inflammatory) or M2 (anti-inflammatory) polarisation. During liver injury, Kupffer cells are activated to release mediators such as TGF-β and platelet-derived growth factor (PDGF), which regulate the activation of HSCs into myofibroblasts, promoting hepatic fibrosis [19]. 

**Figure 2:**
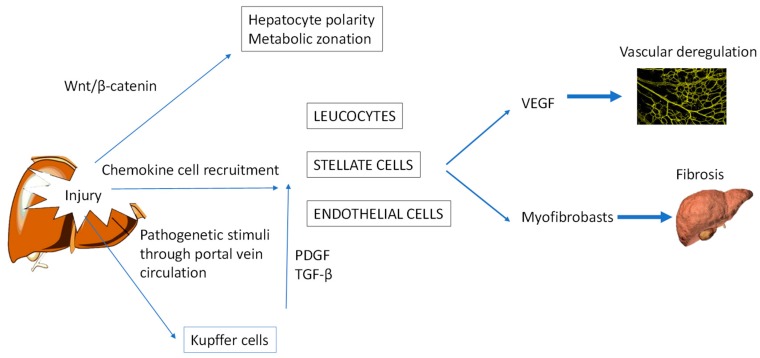
Crosstalk among mediators of liver injury. In the absence of Wnt ligands, the Wnt/β-catenin signalling pathway is mostly inactive. During cell regenerative processes and in certain pathological conditions, the Wnt/β-catenin signal is activated. Wnt/β-catenin signalling controls several processes such as cell growth, differentiation and polarisation. Chemokines released by Kupffer cells during liver injury recruit several cell types such as leukocytes, hepatic stellate cells (HSCs) and endothelial cells to the site of injury. These cells regulate the extracellular VEGF-mediated processing of vascular permeability and angiogenesis and, through the differentiation of HSCs to myofibroblasts, promote fibrosis. Kupffer cells become activated when they phagocytise blood-borne pathogens transported by the portal circulation. Current evidence suggests that these cells are the principal effectors of fibrogenic PDGF- and TGF-β-mediated signalling, which activate HSCs and regulate extracellular matrix accumulation. VEGF, vascular endothelial growth factor; PDGF, platelet-derived growth factor; TGF-β, transforming growth factor beta.

The liver, due to its anatomical location and structure, participates in the induction of tolerance to food. However, after activation of an immune response and inflammatory processes, for example to a neoplasm, hepatic macrophages and HSCs release large amounts of cytokines and adhesion molecules. These proteins contribute to the activation of endothelial cells, which facilitates the extravasation of neoplastic cells into the liver. These cells, in context of the tolerant liver environment, can develop into metastases (Figure 2) [7,9,20,21]. Indeed, metastasis is the result of a complex interaction involving the extracellular matrix, the tumour, the inflammatory cells and the blood vessels. Inflammatory cells produce several types of chemokines at the site of inflammation, thus recruiting additional leukocytes, HSCs and endothelial cells that are overall involved in the homing and proliferation of cancer cells. Changes in the types and concentrations of these chemokines or in the expression of their receptors have been implicated in the development of fibrosis and HCC [22,23].

The structural unit that supports the architecture of each liver lobule is the hepatocyte, which must be able to regenerate and maintain polarity. Hepatocytes are involved in many vital functions such as synthesis, metabolism of carbohydrates, protein and lipids, and removal of toxic substances. For these activities, hepatocytes have three distinct plasma membrane domains separated by tight junctions: basal, lateral and apical [24]. The basal domain faces the hepatic sinusoids and filters the sinusoidal blood flow. The apical domain forms the hepatocyte-bile canalicular network that transports bile acids from blood through hepatocytes into the bile ducts. The lateral membrane domain forms a *zonula occludens* that separates the bile duct from the blood, thus forming a blood–bile barrier that restricts the mixing of blood and bile. Thus, preservation of normal membrane polarity is vital for hepatocyte functionality, such as apical bile secretion and simultaneous basal secretion of large quantities of serum proteins into the blood. The main factor involved in the formation and maintenance of hepatocyte polarisation is the Wnt/β-catenin signalling pathway (Figure 2). Wnt is a secreted glycoprotein that, by binding its receptor, stabilises β-catenin such that the latter protein cannot be phosphorylated. β-catenin then accumulates in the cytoplasm and then translocates to the nucleus, where it activates the cell polarity signalling that regulates the cytoskeleton responsible for the form and polarity of hepatocytes [25]. The concentration of Wnt thus regulates hepatocyte metabolic zonation and polarity. 

## 3. The Liver as an Immunological Organ

The liver is a common site of metastasis for many tumours. This reality is due to the organ’s sinusoidal structure, which permits the extravasation of tumour cells, and also due to its tolerogenic nature as an organ tasked to metabolise gut-derived microbial products and nutrients. Because of these functions, the liver environment favours immune tolerance to counterbalance the impact of stress, which is particularly strong in the liver. Indeed, high concentrations of antigens produced by food transition and the presence of commensal bacteria in the intestine, which are poured in the hepatic blood circulation, especially in cases of inflammatory bowel disease, strongly stimulate the immune system and may cause liver inflammatory [26,27]. 

The hypothesis that the immune system could play a role in the development of neoplasia has been a subject of debate for many years. Evidence in support of the liver tolerance ability includes the observations that immune cells, in particular natural killer (NK) cells, contribute to improved survival after liver transplantation, and the finding that a decrease in immune defences, due to old age, immunosuppressive therapy or autoimmune disease, increases the risk of liver metastasis [28,29,30]. Even smoking, alcohol abuse and poor nutritional status, which can alter the immune system, increase the risk of HCC. Furthermore, neoplasms in immunocompromised subjects are generally more aggressive than in immunocompetent persons. Therefore, a patient’s immunological status has been proposed as a prognostic marker for HCC [31,32].

The liver contains numerous adaptive and innate immune cells that recognise foreign substances, recruit leukocytes, and are present to lymphocytes the antigens taken up from the circulation. In order to be effective, the immune system needs to be activated only against foreign molecules and not activated against host molecules; to do this, the immune response needs to be continuously controlled. The control between activating and inhibiting signals from the immune response is what characterises the liver as an immunologic organ [33]. The liver, through the blood circulation, continuously exchanges immunological information with body tissues. In particular, it secretes cytokines (e.g., interferon gamma (IFN-γ) and interleukins (IL) (IL-2, IL-7 and IL-15)) in response to inflammatory signals mediated by other cytokines, toll-like receptors, tumour necrosis factor alpha (TNF-α). Thus, liver is a crucial organ for host immunity. This exchange of information is facilitated by the extensive filtering of blood by the liver. The total blood volume circulate through the liver about 360 times each day and the liver receiving about 30% of the total blood volume each minute.

**Figure 3:**
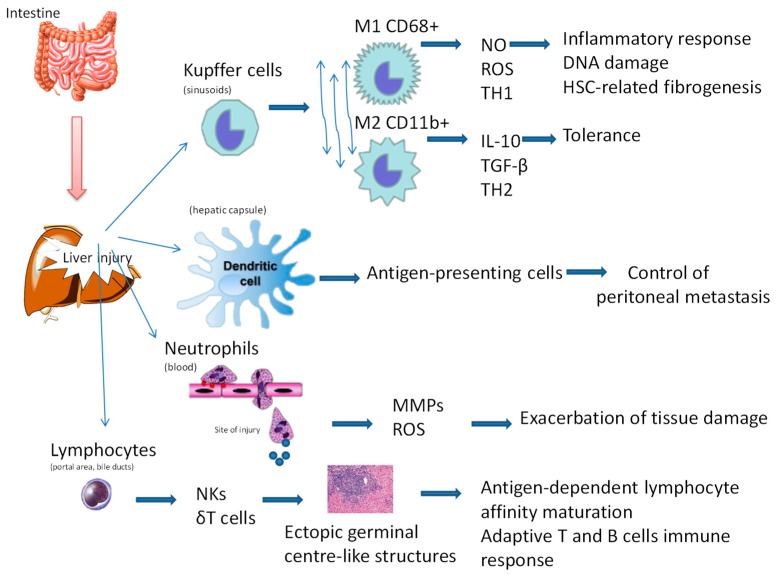
Schematic of the liver harbouring various immune cell populations with different functions. Kupffer cells near or inside the sinusoids decrease immune reactions by releasing cytokines; if these cytokines promote inflammation, the Kupffer cells are acting as M1 macrophages; if the cytokines decrease inflammation and promote tissue repair, the cells are M2 macrophages. Dendritic cells, the main antigen-presenting cells of the immune system, are mostly present in the subcapsular space. The capsule is in contact with the viscera and peritoneum, and thus is a barrier for pathogens and neoplastic cells arriving from these sites. Neutrophils, the most abundant cell type in the blood, accumulate at sites of injury and release matrix metalloproteinases (MMPs) and reactive oxygen species (ROS), exacerbating tissue damage. Lymphocytes in the blood and bile are involved in innate and adaptive immunity and are related in the formation of ectopic germinal centre-like structures in the liver during certain pathological situations (e.g., hepatitis C virus infection). NK (natural killer) cells, natural killer cells; γδ T cells, gamma delta T cells that have a distinctive T cell receptor (TCR) composed of γδ.

The liver harbours several immune cell populations, each with its own specific function and hepatic location (Figure 3) [34]. Kupffer cells invade the space of Disse. They are in direct contact with the blood circulation through microvilli that penetrate the endothelium and interact with hepatocytes. There are two distinct subpopulations of Kupffer cells: an M1 CD68+ subset with phagocytic capacity and an M2 CD11b+ subset with cytokine-producing capacity [35]. The M1 subset produces high levels of nitric oxide (NO) and reactive oxygen species (ROS) that kill microorganisms and skew T cell responses towards a T helper type 1 (TH1) response. The M2 subtype secrets large amounts of IL-10, which is crucial for the induction of tolerance to hepatocyte antigens, and small amounts of IL-12. Moreover, the M2 cells express both pro-inflammatory cytokines (TNF-α, IL-1 and IL-6), especially when exposed to immunocomplexes or bacterial lipopolysaccarides, and anti-inflammatory cytokines (IL-10 and TGF-β). In addition, Kupffer cells have been shown to cross-present antigens to T cells and to recruit T helper type 2 (TH2) cells to the liver [36]. 

Activated Kupffer cells have been implicated in hepatocarcinogenesis [35]. During liver injury, activated Kupffer cells secrete multiple inflammatory cytokines, in particular TNF-α and ROS, which induce hepatocyte growth and cause DNA damage, respectively. A large number of cells that accumulate DNA damage can drive carcinogenesis. Furthermore, in liver injury, activated Kupffer cells produce high levels of chemokines (e.g., CCL2 and CXCL10) that recruit immune cells (macrophages and T cells) to the liver, this process is important for liver regeneration. According to the response to mediators in the liver (e.g., inflammatory IFN-γ, lipopolysaccharide or anti-inflammatory IL-4, IL-10 and IL-13), macrophages give rise to pro-inflammatory M1 macrophages or regulatory M2 macrophages. Polarisation towards the M2 macrophage phenotype is associated with hepatic tissue repair and suppression of the adaptive immune system, thus allowing HCC to grow and metastasise [37]. During liver injury, Kupffer cells also play an indirect role in liver repair through the activation of HSCs by cell–cell interactions and the release of cytokines, including macrophage inflammatory protein (MIP)-1, leading to collagen deposition [38]. When the cause of liver insult ceases, Kupffer cells switch their phenotype towards a reparative function. However, if the insult persists, then fibrosis may develop and the tissue may degenerate, activating a hepatocarcinomatous process (reviewed in [39]). The exact role of Kupffer cells in fibrinogenesis and hepatocarcinogenesis remains elusive, but it is hoped that a better understanding of the functions of different Kupffer cell subsets could help develop new treatments for liver disease [40]. Research in mice has already shown that Kupffer cells can be transplanted from healthy donor animals into animals with experimental liver damage where they exert beneficial effects [41].

Dendritic cells (DCs) are specialised antigen-presenting cells that play key roles in the initiation and direction of T cell responses. Antigens circulating in blood and lymph are captured by DCs, and then loaded DCs migrate through the lymph towards the lymph nodes, where they present the antigen to T cells. In the liver, DCs are found in the subcapsular space and around vessels such as the central veins [42]. Their location in the subcapsular space is fundamental to protecting the liver from bacteria, viruses, toxins and metastasizing neoplastic cells from the peritoneal cavity. Finally, in response to a hepatic viral infection, DCs induce and sustain CD8+ T cells against infected hepatic cells. Given DCs’ important role in initiating antiviral T cell immune function, the possibility of targeting antigens to these cells has been proposed as a vaccination strategy against chronic hepatic virus infections [43]. 

Granulocytes, neutrophils and eosinophils migrate from the blood to the injury site in the liver, following gradients of chemokines from activated immune cells and of fragments of dead cells (e.g., pieces of genomic DNA and mitochondrial byproducts) [44]. These cells accumulate in the microvasculature and then extravasate into the parenchyma by binding to integrin and vascular adhesion molecules. During extensive endothelial cell damage, they directly extravasate without the need for these adhesion molecules. At the site of liver injury, they release proteases including matrix metalloproteinases and generate ROS which exacerbate the tissue damage [45]. In addition to amplifying tissue injury during the acute phase of inflammation, these cells also clear and repair the injured area [46].

Lymphocytes are enriched and naturally activated in the liver [47]. Here, most lymphocytes (up to 65%) are NK cells [48]. Recent studies have suggested that NK cells are involved in HCC development and chronic hepatic viral infection [49,50]. The second most numerous group of lymphocytes in liver are γδ T cells (15%–25% of all T cells) [51]. These cells have T-cell receptors composed of one γ chain and one δ chain, which recognise a limited set of antigens such as stress proteins and non-protein antigens. γδ T cells accumulate in the portal area and bile duct during fibrogenesis, but their role remains elusive [52]. Finally, the liver contains B cells. During infection, intraportal lymphoid follicles have a germinal center-like structure, in which activated B cells are surrounded by a follicular DC and T cell network. The distribution of these B cells and the gene expression patterns in immune cells in the lymphoid follicles around the portal vein are similar to those seen in lymph nodes, suggesting that these structures function as germinal centres [53].

## 4. Role of Liver Damage in Permitting Liver Metastasis

There are several hypotheses regarding the pro-metastatic state of a damaged liver consequent to cirrhosis, steatosis and nonalchoholic fatty liver disease. Some authors sustain that the altered hepatic cytoarchitecture, consequent to cirrhosis and steatosis, creates an unfavourable environment for the development of metastasis [54,55]. Others sustain that these alterations slow the passage of neoplastic cells through the microcirculation, and enhance cellular-vascular contact and accentuate microthrombotic phenomena through the expression of adhesion molecules, thereby facilitating metastasis [56,57,58]. Moreover, inflammation and activation of HSCs during liver injury create a microenvironment favourable not only for the development of primary hepatic tumours but also for the growth of metastases [59]. Neutrophils at an inflammatory site produce numerous molecules involved in inflammation and DNA damage (e.g., ROS), leading to carcinogenesis [60]. Neutrophils are also active in remodelling the extracellular matrix, especially the laminin component, which has been found to trigger cancer cell proliferation [61]. These findings underline the importance of neutrophils in most sites of tumour recurrence and metastasis, and highlight the well-known link between chronic inflammation and cancer.

Steatosis is an accumulation of triglycerides in hepatocytes. Risk factors for steatosis (and steatofibrosis) include metabolic syndrome and hepatitis virus infections. Steatosis is a reversible phenomenon, but when it persists, it modifies the hepatic cytoarchitecture. These changes in the architecture of the liver reduces the flow of blood and blood cells in the liver. Lipid accumulation, combining with ballooning degeneration, chronic portal inflammation, fibrosis, neoplastic microthrombi and an imbalance between the production and reduction of free radical fatty acids all contribute to creating a pro-inflammatory state with altered blood flow. In this setting, a nonalcoholic fatty liver disease (NAFLD) like steatosis develops into nonalcoholic steatohepatitis (NASH), causing fibrosis which results in cirrhosis. Traditionally, HCC was thought to develop mostly from cirrhosis. However, it is now clear that HCC as well as liver metastasis can also develop directly from inflammation without cirrhosis; this disease evolution is associated with metabolic disorders [62,63]. Several studies in animal models showed that obesity, dyslipidaemia and hyperglycaemia predispose to the development of HCC and liver metastases despite the absence of fibrosis, which is mainly present in NASH [64,65,66]. Thus, an increasingly important role is recognised for NAFLD as a leading cause of end-stage liver disease, although other genetic and environmental factors may influence the natural history of HCC. This carcinogenic role is likely due to some characteristics of NAFLD rather than to a fibrosis-induced alteration of hepatic cytoarchitecture (not yet been established in NAFLD). It may be due to obesity with low-grade inflammation, causing steatosis, oxidative stress, lipotoxicity and the secretion of several adipokines, in concert with an insulin-like growth factor signalling [67]. NAFLD in particular, is characterized by a high hepatic triglyceride and cholesterol content. The excessive lipotoxic cholesterol content induces nonalcoholic steatohepatitis, which strongly predisposes to HCC.

NASH is a type of NAFLD characterised by steatosis, inflammation and necrosis. If NASH persists over time, the body’s attempts to repair the damage can lead to fibrosis by the replacement of hepatocytes with connective tissue. NASH can only be diagnosed by liver biopsy, but it can be suspected in patients with obesity, insulin resistance or high serum levels of transaminases; it is associated with a large production of ROS and the risk of HCC [67,68]. 

Oxidative stress, an essential factor in the pathogenesis of gastrointestinal mucosal diseases, was found to occur early in chronic liver diseases and in liver inflammatory responses. Consequently, the extetd of oxidative stress may reflect the degree of liver damage and hence the pro-metastatic predisposition of the parenchyma [69]. Markers of cell injury caused by oxidative stress are 8-oxo-20-deoxyguanosine (8-oxo-dG; a product of DNA oxidation) and malondialdehyde (from the peroxidation of polyunsaturated fatty acids in the plasma membrane). Monitoring these markers in vivo is difficult due to their short half-lives and the balance between oxidative and anti-oxidative species. Thus, today, there is no consensus on the clinical value of assessing oxidative stress. 

## 5. Impact of Intestinal Microbiota on Liver

The intestinal microbiota consists of a variety of bacteria, viruses and fungi, present in different amounts, depending on the site within the intestinal tract and the subject’s age, ethnicity and state of health [70]. The microbiota plays important roles in the development, maturation and functioning of the immune system [71]. It has been suggested that certain microbiotic compositions and the presence of systemic inflammation facilitate HCC carcinogenesis, particularly in patients with cirrhosis and steatosis [72]. 

The interest in gut microbiota has grown recently with the discovery that it interacts with several extra-intestinal organs, including those of the cardiovascular system, the kidneys, brain, bone and especially liver [73]. The composition of the microbiota changes from birth to adulthood under the influence of both intrinsic and extrinsic factors, such as diet, exercise, infections, and antibiotic therapy and surgery [74,75,76]. Several diseases, including obesity, type I diabetes, and the inflammatory bowel diseases like Crohn’s disease and ulcerative colitis, alter the intestinal microbiota [73].

**Figure 4:**
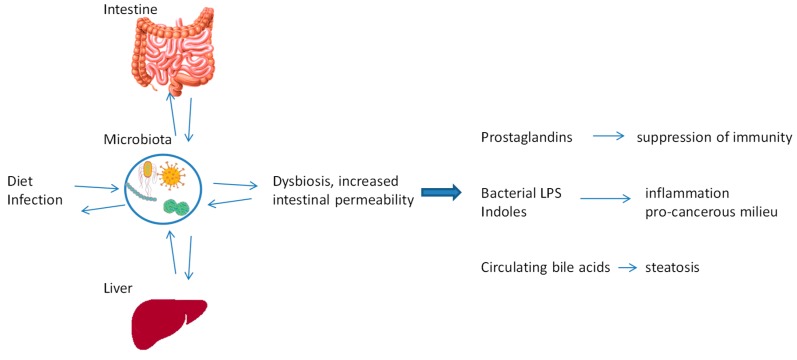
The gut–liver axis. The gut microbiota exacerbates liver diseases by: (i) the production of prostaglandins which suppress tumour immunity; (ii) the translocation of bacterial lipopolysaccharides (LPS) and other metabolites, particularly indoles, to the liver and other organs during states of high intestinal permeability; and (iii) the alterations of bile acid composition, leading to the accumulation of secondary bile acids in the liver.

The liver receives nutrients, toxins and microorganisms via the portal vein from the gut. When the intestine is inflamed, it is more permeable, so a greater amount of toxins and bacteria can reach the liver, favouring the development of inflammation in the liver [77,78,79,80]. Bile acids produced by the liver not only facilitate the absorption and elimination of fats in the intestine, but also interact directly with cellular receptors and bacteria in the intestine. In fact, bile acids and bacteria have complementary effects: bile acids limit the pool of bacteria in the intestine through their bacteriostatic functions, while bacteria reduce the concentration of bile acids by metabolism [81]. Bile acids are implicated in the aetiology of several diseases, including recurrent infection, inflammatory bowel disease, metabolic syndrome and several cancers [77], but how the microbiota change the composition of the bile to cause disease is not clear. Bile acids that do not recycle through the enterohepatic circulation are transformed by gut bacteria into secondary bile acids that return to the liver in conjugated forms [82]. The conjugation process makes the bile acids less toxic, more water-soluble, and capable of protecting against damage from toxic hydrophobic bile acids, which can cause oxidative stress and cell death. Therefore, alterations in the gut microbiota and their metabolism of bile acids could cause hepatotoxicity [83,84]. Gut microbiota can exacerbate liver diseases by several mechanisms (Figure 4): (i) the microorganisms produce prostaglandins that can suppress tumour immunity [85]; (ii) after an increase in intestinal permeability, bacteria and their metabolites (e.g., lipopolysaccharides and indoles) translocate to the liver where they induce inflammation and favour the development of an pro-cancerous milieu [86]; (iii) during high-fat diets, bacteria may alter the bile acid composition, leading to the accumulation of secondary bile acids in the liver [77]. Given this insight into the impact of gut microbiota on liver, there is now interest in treating liver disease by manipulating the microbiota. Dietary interventions being tried include the administration of prebiotics, probiotics or synbiotics, to reduce the serum levels of triglycerides, cholesterol and lipopolysaccharides, combat glucose intolerance and metabolic endotoxemia, and prevent the accumulation of secondary bile acids [85,87,88]. In this context, faecal microbiota transplantation is being explored as a way to optimise microbiota composition and functionality and ultimately treat liver diseases [89,90,91,92,93,94].

## 6. Circadian Homeostasis of Liver Metabolism

The circadian rhythm modulates most cellular functions in relation to the light–dark and temperature cycles caused by rotation of the Earth. The circadian regulation of metabolism also affects carcinogenesis, including that of the liver, as shown in a study in mice [95]. A chronic alteration of the circadian rhythm was found to induce spontaneous HCC in wild-type mice following NAFLD, NASH and then fibrosis [95]. A similar cascade of events often takes place in human obesity. Induction of NAFLD after disruption of the circadian rhythm in mice leads to an increase in triglycerides and free fatty acids. This phenomenon is due to a deregulation of fatty acid entry into mitochondria and to their oxidation to produce energy. As a consequence, high levels of glucose and insulin persist in the blood, and glycogen storage in the liver decreases. This is accompanied by the development of metabolic syndrome (hepatomegaly, bile duct and hepatocyte proliferation, liver inflammation and fibrosis). In summary, chronic alteration of the circadian rhythm altered liver metabolism and caused insulin resistance that, by accelerating cytoplasmic glycolysis, promotes lipid synthesis and storage in liver. However, these metabolic changes occured together with the production of high amounts of ROS. Because ROS induce liver damage and increase cell division, they favour carcinogenesis. This effect is called the “Warburg theory of cancer” [96].

According to the Warburg theory of cancer, cancer cells tend to use anaerobic glycolysis (glucose fermentation) for metabolism instead of the more effective oxidative phosphorylation, because of insufficient cellular respiration in damaged mitochondria. Oxidative phosphorylation produces up to 36 ATP molecules per molecule of glucose, while anaerobic glycolysis results in just two ATP molecules. Consequently, neoplastic cells need an increased glucose uptake from the microenvironment and secrete more lactic acid, the end product of glycolysis. Lactic acid upregulates the expression of hypoxia-inducible factors (HIFs) and VEGF, which stimulate neo-angiogenesis [97]. 

The lactate acidification of the microenvironment also has an immunosuppressive action favouring tumour immune escape [98]. Extracellular acidosis (pH 6.8) was found to inhibit monocyte differentiation and to promote T cell death and the release of cytokines from cytotoxic T cells [99]. Indeed, activated T cells that accumulate in a tumour site cannot perform glycolysis, which is necessary for generating energy for daily functions including cytokine release, due to the blockade of their lactate efflux into the microenvironment as a consequence of an unfavourable ratio between intra- and extracellular pH values. As a consequence, the cytotoxic immune response is blocked. To resolve this problem, at least in part, new drugs that regulate the pH and correct the cellular metabolism are now being developed [100,101].

**Figure 5:**
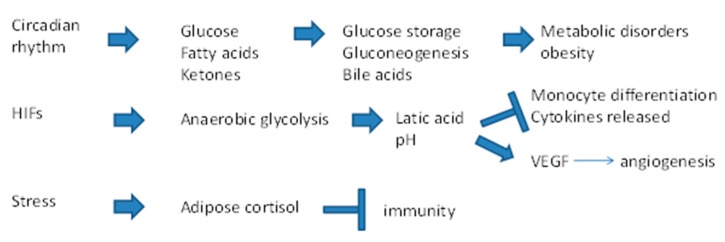
Possible causes of liver damage due to circadian rhythm alterations, hypoxia and psychological stress. Disruption of the circadian rhythm is a risk factor for obesity, metabolic disorders and HCC. Hypoxia-inducible factors (HIFs) control gene expression during hypoxia, enabling cells to survive in a hypoxic environment. HIFs reprogramme the cell’s metabolism towards anaerobic glycolysis to increase energy through ATP production, thus lowering the pH value of tissue. Low pH activates the inflammatory program of monocytes, reduces the number and function of T cells, and promotes angiogenesis. Chronic psychological stress leads to a cortisol secretory burst, which creates an immunosuppressive state in the liver.

Circadian rhythm also influences the complex neuronal network and the release of neurohumoural factors (e.g., cortisol) (Figure 5). It has long been recognised that psychosocial conditions affect the progression of some cancers [102]. Recently, neuronal mechanisms have been implicated in the regulation of proliferation and migration of different tumours and metastases, HCC included [103]. Cortisol is a hormone released into the blood by the adrenal glands under control of the pituitary gland; cortisol release has a diurnal cycle but it is also released in response to stress and low hypoglycemia. Cortisol induces an increase in glycemia by stimulating gluconeogenesis in the liver. Cortisol is also generated by adipose tissue and secreted, together with other hormones, into the blood from where they are taken up by the liver. Thus, psychologicalstress, particular in obese persons, may result in the accumulation of cortisol in the liver, where it contributes to liver steatosis and predisposes to HCC [104,105]. 

## 7. Conclusions

In recent years, the incidence and development of HCC has changed substantially due to the introduction of an anti-hepatitis B virus (HBV) vaccine and anti-HCV therapy. However, there has been no decline in the incidence of HCC associated with other diseases (in particular NAFLD, NASH and metabolic syndrome). A difference concerning HCC occurring in a background of or without cirrhosis also is emerging as a reflection of a difference in HCC pathogenesis. The common denominator of HCC risk among these different diseases, is the development and maintenance of a hepatic inflammatory state, in response to a viral infection, the accumulation of fat, or irritating molecules that reach the liver via blood and bile circulations. The fact that this inflammatory or pro-inflammatory state develops by different mechanisms extends the strategies that can be used at least theoretically, to reduce the risk of recurrence or metastasis. Of particular interest is the alteration of the cytoarchitecture and the microcirculation of the liver associated with the development of fibrosis, an increase in the inflammation of the hepatic microenvironment resulting, in particular, from a greater intestinal permeability to bacteria and toxins and the lipotoxicity associated with the metabolic syndrome, the immunosuppressive status occurring in the hepatic environment deriving from social stress, secretion of adipokines, alteration of the bile acid and the liver metabolism, which favour the engraftment of HCC. Of noteworthy the same molecular, metabolic and cellular processes, appears to favour also the development of recurrence and metastasis in the liver. The definition of specific pathways leading to liver damage offers the possibility of exploring therapeutic or prevention strategies to modulate the risk of disease recovery. Many of the conditions presented in this review could be modified by personalised therapeutic plans aimed at a personalised study of nutritional programs, the glycemic and lipid control, the modification of the intestinal flora and the increase of host immunocompetence, in addition to the obvious and already obsolete treatment of hepatic viral infections. Moreover, targeted therapies on distinct metabolic pathways involved in the progression of liver damage are now in development as reviewed in [106]. Promising drugs being currently studied are, for examples, those targeting the squalene epoxidase which is involved in the biosynthetic pathway leading to cholesterol. The squalene epoxidase was found to accumulate in the liver affected by NAFLD, and is associated with poor survival in patients with HCC [107] and analogues of an enzyme of the classic pathway of bile acid synthesis from cholesterol, the fibroblast growth factor 19, which are demonstrated to reduce liver-fat content, inflammation and fibrosis in patients with NASH [108,109].
